# Exploiting the Warburg Effect: Co‐Delivery of Metformin and FOXK2 siRNA for Ovarian Cancer Therapy

**DOI:** 10.1002/smsc.202300192

**Published:** 2024-01-29

**Authors:** Wenhui Zhou, Xiaodong Ma, Jianpeng Xiao, Xiaohui He, Chang Liu, Xiaoyu Xu, Tapani Viitala, Jing Feng, Hongbo Zhang

**Affiliations:** ^1^ Shanghai Fengxian District Central Hospital Shanghai 201499 China; ^2^ Pharmaceutical Sciences Laboratory Åbo Akademi University 20520 Turku Finland; ^3^ Turku Bioscience Centre University of Turku and Åbo Akademi University 20520 Turku Finland; ^4^ The Third School of Clinical Medicine Southern Medical University Guangzhou 510630 China; ^5^ School of Laboratory Medicine and Biotechnology Southern Medical University Guangzhou Guangdong Province 510515 China; ^6^ Department of Laboratory Medicine & Central Laboratory Shanghai Fengxian District Central Hospital Shanghai 201499 China; ^7^ Longgang District People's Hospital of Shenzhen Shenzhen 518172 China; ^8^ Drug Research Program Division of Pharmaceutical Chemistry and Technology Faculty of Pharmacy University of Helsinki 00014 Helsinki Finland

**Keywords:** FOXK2 siRNA, metformin, microfluidic technology, ovarian cancer therapy, Warburg effect

## Abstract

Ovarian cancer remains a significant health issue worldwide, often facing limitations in treatment due to side effects and drug resistance. Tumor cells typically undergo the “Warburg effect,” preferring glycolysis, which leads to their rapid growth and survival. Metformin, a widely used diabetes medication, targets 5' adenosine monophosphate‐activated protein kinase (AMPK), reducing glycolysis and thereby slowing tumor growth. Additionally, forkhead box protein K2 (FOXK2), a transcription factor often found in excess in many tumors, promotes glycolysis and tumor development. Delivering metformin and FOXK2 siRNA directly to the tumor site in the body is challenging due to the metformin's poor water solubility and the fragile nature of siRNA. To address this, zirconium and 5,10,15,20‐tetra(4‐pyridyl)porphyrin nanoparticles loaded with FOXK2 siRNA, enveloped in cell membrane, co‐encapsulated with metformin in gelatin methacrylate microspheres (ZrTCP@siFOXK2@CM/Met@GelMA) hydrogel microspheres are developed for effective dual delivery. These microspheres facilitate targeted drug delivery, photothermal therapy with near‐infrared light, and interference with glucose metabolism. These results show that infrared light combined with metformin and FOXK2 siRNA successfully activates the AMPK pathway, reducing ovarian cancer growth. This method offers a promising new direction in treatment, utilizing the complex metabolic characteristics of ovarian cancer to achieve better results.

## Graphical abstract

An innovative therapeutic approach leverages the Warburg effect in ovarian cancer, combining forkhead box protein K2 (FOXK2) siRNA and metformin. Using advanced microfluidic technology, zirconium and 5,10,15,20‐tetra(4‐pyridyl)porphyrin nanoparticles loaded with FOXK2 siRNA, enveloped in cell membrane, co‐encapsulated with metformin in gelatin methacrylate microspheres (ZrTCP@siFOXK2@CM/Met@GelMA) hydrogel microspheres, are introduced, highlighting a transformative strategy to target cancer's metabolic vulnerabilities effectively.

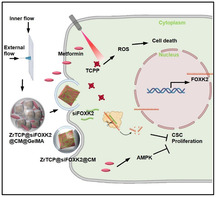

## 1 Introduction

Ovarian cancer ranks among the deadliest gynecological malignancies, casting a significant shadow over women's health globally.^[^ ^1^ ^]^ Despite the marked progress in surgical procedures and supplementary treatments, handling this malignancy continues to be a daunting task. The key challenges include the adverse side effects from therapeutic interventions and the inexorable emergence of drug resistance during chemotherapy regimens.^[^ ^2^, ^3^, ^4^, ^5^, ^6^ ^]^ This underscores the urgent need for innovative therapies that accurately target the core processes behind tumor growth and survival.

The unique metabolism of tumor cells, mainly known for glycolysis or the “Warburg effect,” has always intrigued cancer researchers.^[^ ^7^ ^]^ Within this metabolic shift, cells predominantly lean toward glycolysis over oxidative phosphorylation, even in oxygen‐rich environments. This grants cancer cells distinct advantages, encompassing swift proliferation, heightened metastatic propensities, and resilience against programmed cell death.^[^ ^8^, ^9^ ^]^ At the heart of this metabolic tale stands 5' adenosine monophosphate‐activated protein kinase (AMPK), the guardian of cellular energy equilibrium. Attuned to cellular energy variations, AMPK deftly calibrates both glycolytic and mitochondrial processes, maintaining a balanced energetic state.^[^ ^10^, ^11^ ^]^ AMPK's role in thwarting tumorigenic trajectories and its suppressive impact on cell growth and protein genesis is well established.^[^ ^12^, ^13^ ^]^ This narrative spotlight shifts to metformin, a preeminent medication for type 2 diabetes. Exemplifying notable anticancer virtues, metformin's therapeutic principle hinges on AMPK activation, tempering the glycolytic enthusiasm intrinsic to tumor cells. By modulating AMPK dynamics, metformin not only curtails protein synthesis and cell proliferation but also rebuffs the glycolytic axis, underscoring its potent candidacy in cancer combat.^[^ ^14^, ^15^, ^16^, ^17^ ^]^

Interlaced within this metabolic tapestry is the transcription factor forkhead box protein K2 (FOXK2). With conspicuous overexpression across varied tumors, FOXK2 emerges as a pivotal nexus in glycolytic oversight.^[^ ^18^ ^]^ Cutting‐edge studies underscore FOXK2's commanding role in steering enzymes and pathways integral to glycolysis, affirming its stature as both a glycolytic champion and a catalyst for tumorigenesis.^[^ ^19^, ^20^ ^]^ This revelation promotes a rejuvenated lens on FOXK2, beyond just a diagnostic lighthouse, positioning it as a promising therapeutic target. In light of metformin's robust glycolytic inhibition and FOXK2's glycolytic affinities, a harmonized approach looms on the horizon. Melding metformin's action with FOXK2 silencing offers a tantalizing prospect, potentially unveiling a transformative paradigm in targeting the glycolytic trajectory for refined cancer therapy.

However, despite the optimism surrounding recent advances, significant hurdles persist in the targeted delivery of metformin and siFOXK2 RNA for maximizing cancer treatment efficacy. While metformin, in tandem with chemotherapy drugs, has shown promising therapeutic outcomes,^[^ ^21^, ^22^, ^23^ ^]^ the required cytotoxic concentration for cancer cells remains prohibitively high. Achieving this optimal concentration in tumor tissues through standard means, like intravenous injections, is daunting. Conversely, siRNAs, such as siFOXK2 RNA, with their transformative potential in gene silencing, grapple with multiple challenges.^[^ ^24^, ^25^, ^26^ ^]^ The innate instability of siRNA molecules stands out as a primary concern. Compounding this is the biological membrane barrier, which restricts the efficient delivery of these sequences into target cells, a predicament more pronounced within the intricate tumor microenvironments. Consequently, this barrier has become a significant roadblock for the broader adoption of nucleotide‐based treatments.^[^ ^27^ ^]^ Therefore, crafting a precise co‐delivery system for metformin and FOXK2 siRNA is crucial for tapping into their synergistic therapeutic potential against cancer.

Capitalizing on the synergistic therapeutic benefits of FOXK2 siRNA and metformin, we employed microfluidic technology to craft the zirconium and 5,10,15,20‐tetra(4‐pyridyl)porphyrin nanoparticles loaded with FOXK2 siRNA, enveloped in cell membrane, co‐encapsulated with metformin in gelatin methacrylate microspheres (ZrTCP@siFOXK2@CM/Met@GelMA) hydrogel microspheres, heralding a new paradigm in enhanced treatment.^[^ ^28^, ^29^ ^]^ Central to this pioneering approach is the GelMA substrate, lauded for its outstanding biocompatibility and precision in drug release.^[^ ^30^ ^]^ This deliberate integration is crucial in overcoming the inherent barriers of reaching optimal therapeutic concentrations of metformin within tumor tissues, a feat seldom achieved through conventional delivery methods.^[^ ^31^ ^]^ By engineering GelMA microspheres tailored for intratumoral administration, we champion localized drug delivery, curtailing systemic side effects and bolstering therapeutic efficacy.^[^ ^32^ ^]^ Encapsulated within these microspheres are both metformin and the ingeniously designed ZrTCP@siFOXK2@CM nanoparticles (NPs). These NPs represent a confluence of the metallic zirconium (Zr) and the photosensitizing agent 5,10,15,20‐tetra(4‐pyridyl)porphyrin (TCPP), giving rise to the versatile ZrTCP metal‐organic frameworks (MOFs).^[^ ^33^ ^]^ Characterized by their porous construct, these MOFs excel in siRNA encapsulation and are further sheathed with a tumor‐cell‐sourced membrane, enhancing their tumor‐targeting ability.^[^ ^34^, ^35^, ^36^ ^]^ But their ingenuity stretches further. At the heart of their design lies the TCPP component, which, when stimulated by near‐infrared light, triggers a photodynamic reaction, releasing reactive oxygen species (ROS) within the tumor milieu.^[^ ^37^, ^38^ ^]^ This cascade of ROS serves a twofold purpose: direct cytotoxic effects causing cellular membrane damage and consequent cell death, and strategic interference with glucose metabolism.^[^ ^39^, ^40^, ^41^, ^42^ ^]^ Such intervention enhances the precision targeting of metformin and FOXK2 siRNA on these metabolic pathways, accentuating their collective antitumor prowess.

The synthesized ZrTCP NPs, with impressive dispersity in ethanol solution, presented an approximate particle size of 104.5 ± 8.4 nm. The tumor‐cell‐membrane‐coated NPs showcased high cellular uptake efficiency and superior lysosome escape potential. In conjunction with metformin, ZrTCP@siFOXK2@CM NPs exhibited heightened cytotoxicity, especially under infrared light, significantly impacting SKOV3 and OVCAR3 ovarian cancer cells. Upon exposure to infrared light, these NPs notably raised cellular ROS levels, and their synergistic use with metformin prominently activated the AMPK signaling pathway in SKOV3 cells. In in vivo studies, the GelMA microspheres, ZrTCP@siFOXK2@CM/Met@GelMA, demonstrated impressive tumor tissue retention and significant tumor suppression under laser irradiation. In sum, our work introduces a pioneering therapeutic strategy, leveraging the metabolic vulnerabilities of ovarian cancer for enhanced treatment outcomes.

## 2 Results

### 2.1 FOXK2 Silencing‐Induced Cell Death in Ovarian Cancer is Enhanced by Metformin

**Figure 1:**
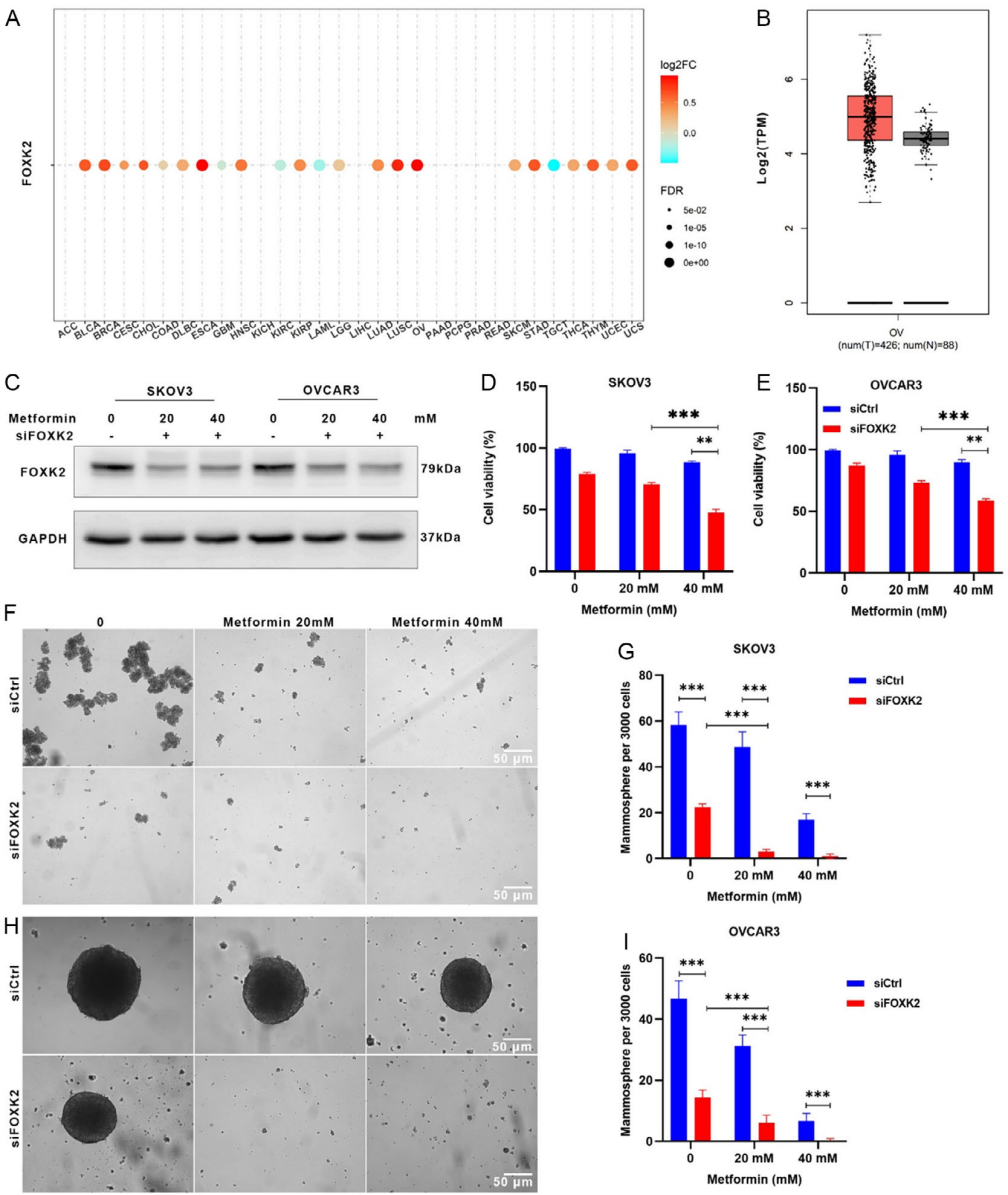
Metformin intensified the inhibitory effect of FOXK2 siRNA on the proliferation and mammosphere formation of SKOV3 and OVCAR3 cells. A,B) FOXK2 expression across Cancer Genome Atlas (TCGA) cancers (A) and ovary cancers (B) from TCGA database (*P* < 0.05). C) Expression of FOXK2 in SKOV3 and OVCAR3 cells tested by western blot assay; cell viability of D) SKOV3 and E) OVCAR3 cells after treatment of siFOXK2 and metformin for 48 h tested by Cell Counting Kit‐8 (CCK‐8) assay (*n* = 3). Data are presented as the mean ± standard deviation (SD) from three independent experiments. F–I) Representative images and quantification of mammospheres formed from SKOV3 (F,G) and OVCAR3 (H,I) cell lines after treatment of siFOXK2 and metformin for 24 h (scale bar = 50 μm). Statistical significance among groups in (D,E,G,I) was determined by one‐way analysis of variance (ANOVA), *P < 0.05, **P < 0.01, ***P < 0.001, and ****P < 0.0001.

To explore the role of the FOXK2 gene in ovarian cancer, we analyzed its expression in 30 types of pan‐cancer and adjacent non‐tumor tissues using The Cancer Genome Atlas (TCGA) database. Our results (**Figure** **1**A) revealed a significant overexpression of FOXK2 in ovarian cancer tissues, with markedly lower expression in the adjacent non‐tumor tissues. This differential expression pattern indicates that FOXK2 may serve as an oncogene in ovarian cancer and suggests that it could be a potential therapeutic target. The broad pan‐cancer analysis contextualized these findings, reinforcing the specificity of the role of FOXK2 in ovarian cancer within the larger oncological landscape (Figure 1B).

To further elucidate the functional significance of FOXK2 in ovarian cancer, we silenced its expression in SKOV3 and OVCAR3 cells using siRNA. Post transfection, we observed a pronounced decrease in cell viability (Figure 1C–E) and tumor sphere‐forming ability (Figure 1F–I), reflecting the importance of FOXK2 in ovarian cancer progression. Subsequent incubation with 20–40 mm of metformin further weakened the cells’ survival and sphere‐forming ability. These findings highlight a potential synergistic effect between FOXK2 silencing and metformin, offering insights into novel therapeutic strategies for ovarian cancer.

Collectively, our study illuminates the pivotal role of FOXK2 in the progression of ovarian cancer. By demonstrating its overexpression in tumors and its functional importance in cell survival and growth, we have identified FOXK2 as a promising therapeutic target. The synergistic effects of FOXK2 silencing and metformin treatment lay the groundwork for innovative treatment modalities in ovarian cancer. Further studies are required to translate these findings into clinically effective interventions.

### 2.2 Synthesis and Characterization of ZrTCP@siFOXK2@CM NPs

**Scheme 1:**
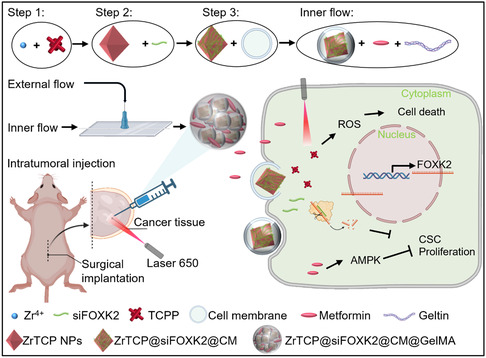
Schematic representation of the preparation process for ZrTCP@siFOXK2@CM/Met@GelMA microgel and its antitumor mechanism.

**Figure 2:**
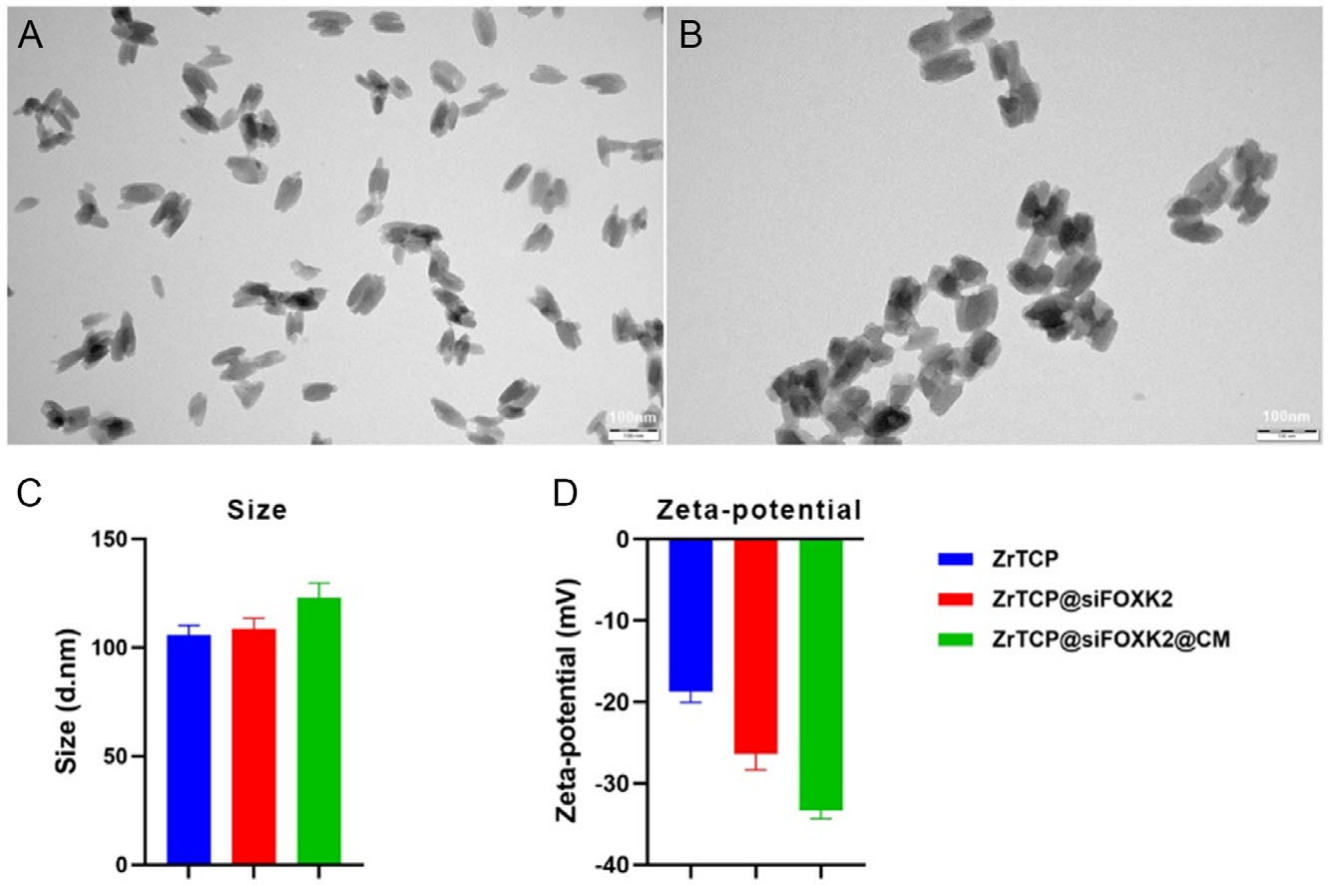
Morphology and characteristics of ZrTCP@siFOXK2 and ZrTCP@siFOXK2@CM nanoparticles (NPs). A,B) Representative images of ZrTCP (A) and ZrTCP@siFOXK2@CM (B) NPs captured by transmission electron microscopy (scale bar = 100 nm); C) size and D) zeta‐potential of ZrTCP, ZrTCP@siFOXK2, and ZrTCP@siFOXK2@CM NPs measured by dynamic light scattering. Data are presented as the mean ± SD from three independent experiments, statistical significance among groups was determined by one‐way ANOVA, *P < 0.05, **P < 0.01, ***P < 0.001, and ****P < 0.0001.

In our study, we ingeniously utilized the photosensitizer TCPP as a ligand and, through a nanoscale self‐assembly process, reacted it with Zr ions to synthesize ZrTCP NPs (**Scheme** **1**). These NPs displayed a fusiform shape, with a diameter of approximately 104 nm and a negative charge (**Figure** **2**A–C). In a subsequent step, we successfully loaded FOXK2 siRNA into these NPs by means of self‐adsorption, thereby creating ZrTCP@siFOXK2 NPs. Interestingly, this procedure did not significantly alter the NP size, but the surface charge was markedly reduced to −27.8 mV. Quantitative analysis revealed that the loading capacity of the ZrTCP@siFOXK2 NPs could reach up to 100 mg g^−1^. To further enhance the protective effect of the NPs for siRNA and targeting specificity to tumor cells, we isolated and purified the cell membrane from SKOV3 cells. Through overnight stirring, we prepared biomimetic NPs with cell‐membrane coating, termed ZrTCP@siFOXK2@CM. These cell‐membrane‐coated NPs maintained an elliptical shape, but exhibited clear membrane structures on their surface (Figure 2B). Significantly, the particle size of the cell‐membrane‐coated NPs increased to 125.3 nm, and the surface charge further decreased to −34.6 mV (Figure 2D).

In conclusion, our fabricated ZrTCP@siFOXK2 NPs, as well as ZrTCP@siFOXK2@CM, possess appropriate size and physical morphology, coupled with a high siRNA loading capacity. Theoretically, these attributes confer feasibility for intracellular siRNA delivery, positioning them as promising vectors for targeted gene silencing applications. These findings provide a novel insight into NP engineering and underscore the potential of ZrTCP@siFOXK2@CM NPs for therapeutic interventions in oncological research.

### 2.3 Cellular Interaction of ZrTCP@siFOXK2@CM NPs and Ovary Cancer Cells

**Figure 3:**
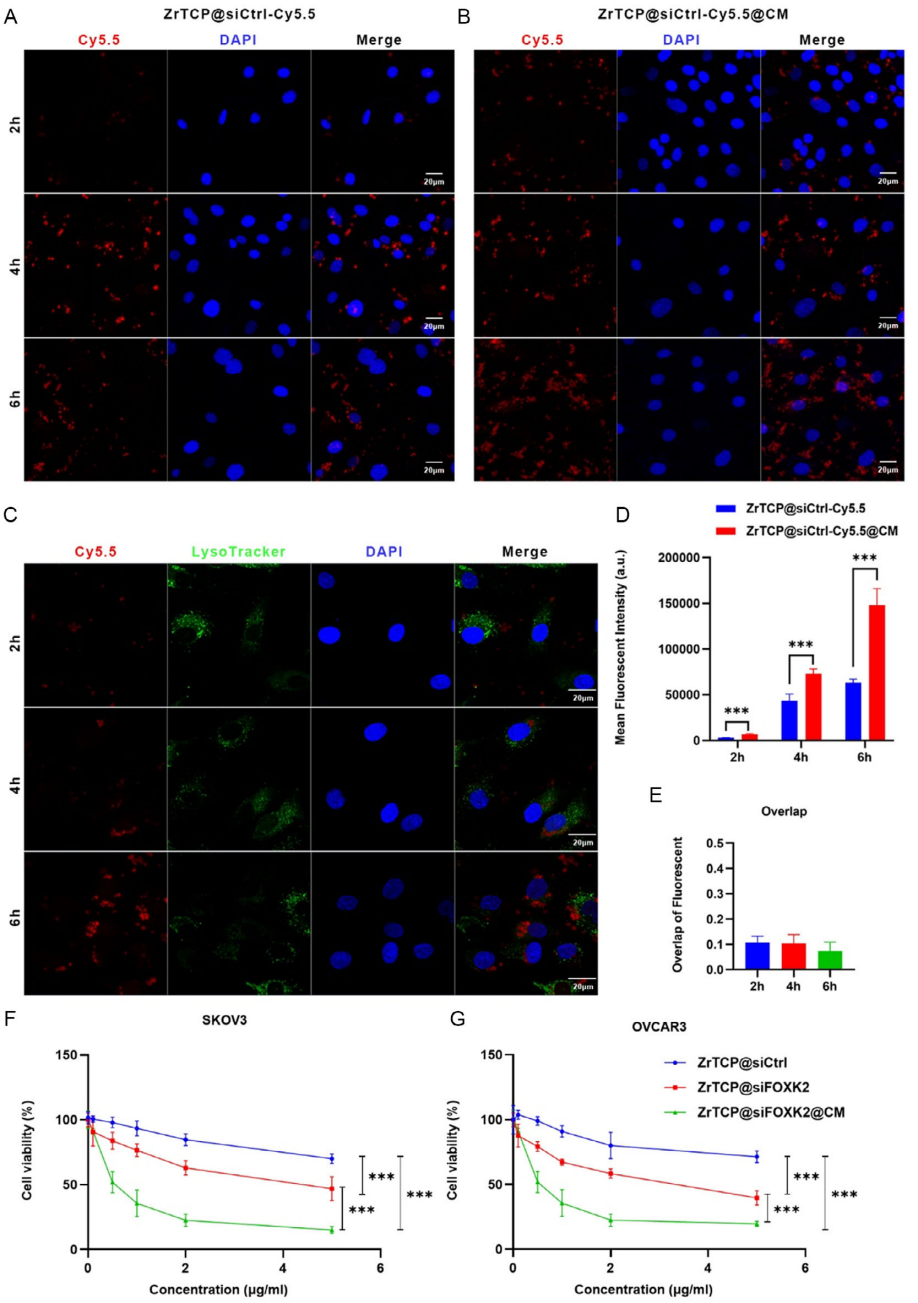
Confocal imaging, fluorescence intensity, and cellular viability of SKOV3 and OVCAR3 cells post‐NP uptake. A,B) Representative confocal microscopy images of SKOV3 cells after uptake of ZrTCP@siCtrl‐Cy5.5 (A) and ZrTCP@siCtrl‐Cy5.5@CM (B) NPs (scale bar: 20 μm). C) Representative confocal microscopy images of ZrTCP@siCtrl‐Cy5.5@CM NPs colocalized with lysosomes after endocytosis (red: ZrTCP@siCtrl‐Cy5.5@CM NPs; green: lysosome; blue: nucleus; scale bar: 20 μm). D) Quantitative data on the mean fluorescent intensity of SKOV3 cells following uptake of ZrTCP@siCtrl‐Cy5.5 and ZrTCP@siCtrl‐Cy5.5@CM NPs (*n* = 3). E) Overlap of the fluorescent signal from ZrTCP@siCtrl‐Cy5.5@CM NPs with that of the lysosome following uptake (*n* = 3). F,G) Cell viabilities of SKOV3 (F) and OVCAR3 (G) cells after treatment of ZrTCP@siCtrl, ZrTCP@siFOXK2, and ZrTCP@siFOXK2@CM NPs for 48 h measured by CCK‐8 assay (*n* = 3). Data are presented as the mean ± SD from three independent experiments, statistical significance among groups was determined by one‐way ANOVA, *P < 0.05, **P < 0.01, ***P < 0.001, and ****P < 0.0001.

Building upon previous results, we sought to further validate the feasibility of ZrTCP@siFOXK2 NPs as siRNA intracellular delivery carriers. To this end, ZrTCP@siCtrl‐Cy5.5 and ZrTCP@siCtrl‐Cy5.5@CM NPs were synthesized using Cy5.5‐labeled control siRNA, and subsequently incubated with logarithmically growing SKOV3 cells over a period of 2–6 h. Observations revealed that both types of NPs exhibited intracellular aggregation as early as 2 h post‐incubation, with a time‐dependent increase in Cy5.5 red fluorescence (**Figure** **3**A,B). Remarkably, cell‐membrane‐coated ZrTCP@siCtrl‐Cy5.5@CM demonstrated enhanced intracellular accumulation compared to its non‐coated counterpart (Figure 3A,B and S4, Supporting Information), supporting the hypothesis that membrane‐coating augments NP uptake and intracellular delivery efficiency.

In a subsequent experiment, we employed green lysosomal fluorescent dye LysoTracker to investigate the lysosomal escape capability of ZrTCP@siCtrl‐Cy5.5@CM NPs within SKOV3 cells (Figure 3C–E). The data revealed minimal colocalization between lysosomal green fluorescence and the red fluorescence of ZrTCP@siCtrl‐Cy5.5@CM, regardless of incubation time (Figure 3D–F). This suggested that ZrTCP@siCtrl‐Cy5.5@CM NPs could either efficiently escape lysosomal compartments or enter cells via pathways independent of the lysosomal route.

Finally, the cytotoxicity profile of ZrTCP@siFOXK2 and ZrTCP@siFOXK2@CM NPs was explored. The findings indicated a notable reduction in cell survival rates to 72.3% and 69.8% for SKOV3 and OVCAR3 cells, respectively, after 48 h incubation at 2 μg mL^−1^ (Figure 3F,G). Moreover, survival rates were further reduced to 20.3% and 25.6% with ZrTCP@siFOXK2@CM, emphasizing its superior inhibitory effect. A caveat emerged with the observation that higher concentrations of ZrTCP@siCtrl exerted significant cytotoxic effects, revealing a potential underlying toxicity of the ZrTCP NPs themselves.

In summary, the current study affirms the promising characteristics of ZrTCP NPs in cellular uptake and lysosomal escape, with cell‐membrane‐coating serving as an effective strategy for enhancing efficiency. Additionally, the significant inhibitory effects of ZrTCP@siFOXK2 NPs on SKOV3 and OVCAR3 cell proliferation were elucidated, with further enhancement observed upon membrane encapsulation. These collective insights underline the potential of ZrTCP NPs for targeted siRNA delivery, indicating their potential applicability in therapeutic strategies for ovarian cancer.

### 2.4 Synthesis and Characterization of ZrTCP@siFOXK2@CM@GelMA Microgel

**Figure 4:**
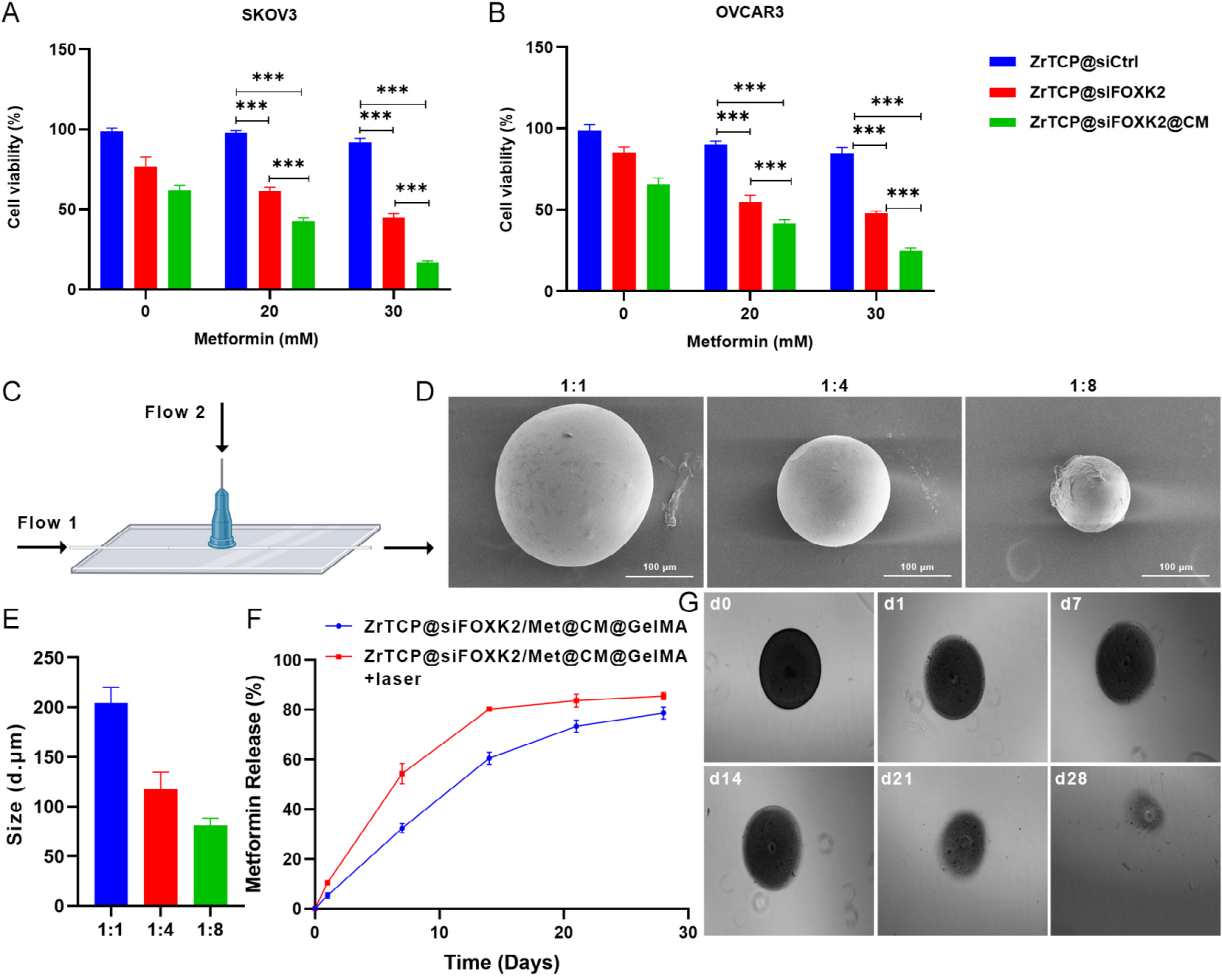
Preparation and characterization of ZrTCP@siFOXK/Met2@CM@GelMA microgel. A,B) Cell viabilities of SKOV3 (A) and OVCAR3 (B) cells after 48 h of treatment with ZrTCP@siCtrl, ZrTCP@siFOXK2, or ZrTCP@siFOXK2@CM NPs combined with metformin, as measured by the CCK‐8 assay (*n* = 3). C) Schematic of microfluidics chip for ZrTCP@siFOXK2/Met@CM@GelMA microgel preparation. D) Representative images of ZrTCP@siFOXK2/Met@CM@GelMA microgel prepared with different flow 1:flow 2 flow speed ratio (scale bar: 100 μm). E) Size of ZrTCP@siFOXK2/Met@CM@GelMA microgel formed at different flow 1:flow 2 flow speed ratio (*n* = 3). F) Metformin release profile of ZrTCP@siFOXK2/Met@CM@GelMA microgel in deionized water with or without 650 nm laser irradiation (0.5 W cm^−2^, 5 min every 3 days) (*n* = 3). G) Representative images showing the degradation of ZrTCP@siFOXK2/Met@CM@GelMA microgel in deionized water. Data in (A,B,E,F) are presented as the mean ± SD from three independent experiments, statistical significance among groups was determined by one‐way ANOVA, *P < 0.05, **P < 0.01, ***P < 0.001, and ****P < 0.0001.

Building on the initial findings, we extended our investigation to determine the synergistic effect of ZrTCP@siFOXK2 NPs with metformin, given that prior studies confirmed the ability of metformin to enhance the inhibitory effect of FOXK2 siRNA on ovarian cancer cells. ZrTCP@siFOXK2 and ZrTCP@siFOXK2@CM NPs were incubated with logarithmically growing SKOV3 and OVCAR3 cells at a concentration of 1 μg mL^−1^, in conjunction with 20–30 mm of metformin. Following 48 h of incubation, a significant reduction in cell survival rates was observed in both ZrTCP@siFOXK2 and ZrTCP@siFOXK2@CM NP‐treated groups relative to the ZrTCP@siCtrl control group (**Figure** **4**A,B). More remarkably, the addition of metformin further significantly decreased the survival rates, with the lowest rates attained in the 30 mm metformin and ZrTCP@siFOXK2@CM combination group, reaching 27.1% and 32.5% for SKOV3 and OVCAR3 cells, respectively. These results strongly indicate that ZrTCP@siFOXK2 NPs and metformin exert a synergistic inhibition on tumor cell proliferation.

Subsequently, in an effort to optimize the administration of ZrTCP@siFOXK2@CM NPs and metformin, enhance drug utilization efficiency, and mitigate potential toxicity, we utilized microfluidic technology to encapsulate ZrTCP@siFOXK2@CM NPs and metformin within GelMA to create injectable, slow‐degrading GelMA microgel beads suitable for in situ tumor injection (Figure 4C). As depicted in Figure 4C, an aqueous solution containing 2 μg mL^−1^ of ZrTCP@siFOXK2@CM NPs, 30 mm metformin, GelMA, and cross‐linking agents was used as the inner phase (Flow 1), and a 5% Span80 mineral oil solution was employed as the outer phase (Flow 2). By manipulating the flow rate ratio between Flow 1 and Flow 2, we successfully fabricated ZrTCP@siFOXK2/Met@CM@GelMA microbeads ranging from 70 to 200 μm in diameter (Figure 4D,E). We selected the 70 μm ZrTCP@siFOXK2@CM/Met@GelMA microbeads for subsequent experiments.

Interestingly, the release profile of metformin from the ZrTCP@siFOXK2@CM/Met@GelMA microgel revealed a continuous release over 28 days, with a maximum release of 78.6% (Figure 4F). This correlated with observable, gradual collapse of the microbeads, culminating in complete degradation at 28 days (Figure 4G). When exposed to 650 nm laser irradiation, the release rate of metformin significantly accelerated, reaching a maximum release of 80.8% after 14 days. Based on these findings, we inferred that the ZrTCP@siFOXK2/Met@CM@GelMA microgel exhibits excellent sustained‐release efficiency for both metformin and ZrTCP@siFOXK2/Met@CM NPs. The enhanced release upon 650 nm laser irradiation is likely attributed to the photosensitive material TCPP within the ZrTCP NPs, which responded to the laser, stimulating release through photodynamic reactions.

In conclusion, this part of the study demonstrated the synergistic antitumor effect of ZrTCP@siFOXK2 NPs with metformin and introduced a novel microgel delivery system, facilitating controlled, sustained release. The promising release characteristics, augmented by light‐triggered responsiveness, underscore the potential of ZrTCP@siFOXK2/Met@CM@GelMA microgels for localized cancer therapy. Further studies will be essential to assess the in vivo efficacy and safety profile of this innovative therapeutic approach.

### 2.5 Laser Irradiation Elevated ZrTCP@siFOXK2@CM@GelMA Microgel‐Mediated Cell Death at Cellular Level

**Figure 5:**
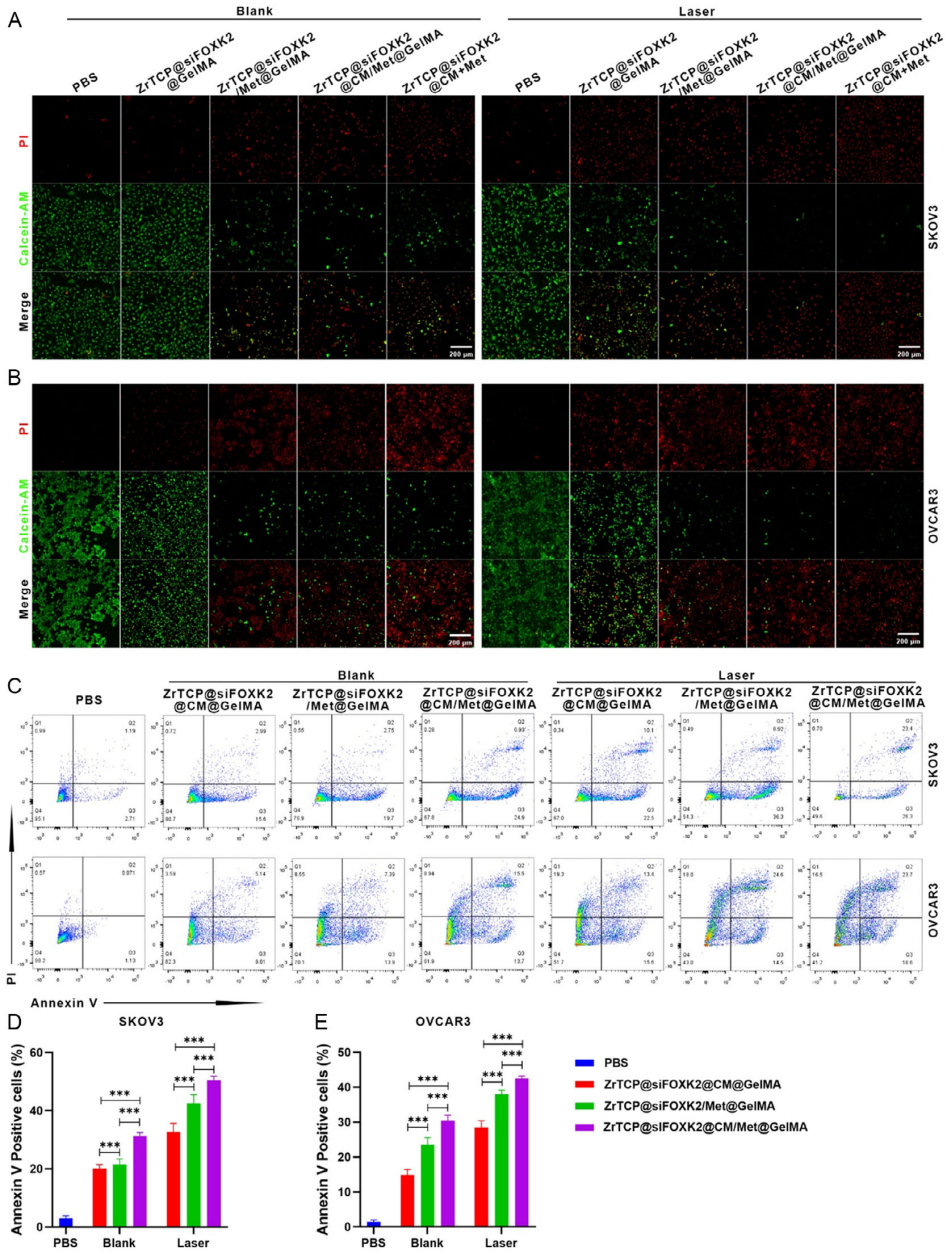
ZrTCP@siFOXK2@CM@GelMA microgel‐induced cell death in SKOV3 and OVCAR3 cells. A,B) Representative confocal microscopy images of SKOV3 (A) and OVCAR3 (B) cells after treatment of PBS, ZrTCP@siFOXK2@GelMA, ZrTCP@siFOXK2/Met@GelMA, ZrTCP@siFOXK2@CM/Met@GelMA, or ZrTCP@siFOXK2@CM + Met for 48 h and combined with/without 650 nm laser irradiation (0.5 W cm^−2^, 5 min) (red: PI; green: calcein AM; scale bar: 200 μm). C) Apoptotic cells and D,E) quantification data after 48 h of treatment, analyzed by flow cytometry. Data in (D,E) are presented as the mean ± the standard error of the mean (S.E.M.) from three independent experiments, statistical significance among groups was determined by one‐way ANOVA, *P < 0.05, **P < 0.01, ***P < 0.001, and ****P < 0.0001.

Continuing from the aforementioned research, we sought to further validate the tumor‐suppressing efficacy of our synthesized GelMA microspheres at the cellular level. By employing techniques such as calcein‐acetoxymethyl ester/propidium iodide (calcein AM/PI) live/dead cell staining and flow cytometric analysis, we examined the apoptosis levels in SKOV3 and OVCAR3 cells under various GelMA microsphere formulations, both with and without laser irradiation. As depicted in the corresponding **Figure** **5**A,B, in comparison with the phosphate‐buffered saline (PBS) group, the cells treated with 1 μg mL^−1^ NPs of ZrTCP@siFOXK2/Met@GelMA, ZrTCP@siFOXK2@CM/Met@GelMA, and ZrTCP@siFOXK2@CM@GelMA + 30 mm free metformin for 48 h displayed a significant increase in red fluorescent PI‐labeled dead cells, with a concomitant substantial reduction in green fluorescent calcein‐AM‐labeled live cells. Following the addition of 650 nm laser irradiation, the SKOV3 and OVCAR3 cells incubated with ZrTCP@siFOXK2GelMA exhibited a marked increase in red fluorescent signals representing dead cells and a significant reduction in green fluorescent signals representing live cells. Moreover, regardless of additional laser exposure, the groups treated with metformin‐containing ZrTCP@siFOXK2/Met@CM@GelMA and ZrTCP@siFOXK2@CM@GelMA +30 mm free metformin showed even more dead cells and fewer live cell signals.

Further deepening our analysis, we utilized flow cytometry to provide additional clarity regarding the antitumor effects of ZrTCP@siFOXK2/Met@CM@GelMA. As demonstrated in Figure 5C–E, following the treatment of SKOV3 and OVCAR3 cells for 48 h with ZrTCP@siFOXK2@CM@GelMA, ZrTCP@siFOXK2/Met@GelMA, and ZrTCP@siFOXK2@CM/Met@GelMA, there was a marked increase in Annexin‐V‐positive apoptotic cell populations. This effect was further amplified under the influence of 650 nm laser irradiation. The ZrTCP@siFOXK2@CM/Met@GelMA + laser irradiation group displayed the highest Annexin‐V‐positive cell populations, measuring 52.3% and 42.6% in SKOV3 and OVCAR3 cells, respectively. In summary, these findings further delineate the cellular‐level antitumor effects of ZrTCP@siFOXK2@CM/Met@GelMA and confirm the synergistic antitumor efficacy when combined with 650 nm laser irradiation.

### 2.6 Biodistribution and in Vivo Safety of ZrTCP@siFOXK2@CM NPs and ZrTCP@siFOXK2/Met@CM@GelMA Microgel

**Figure 6:**
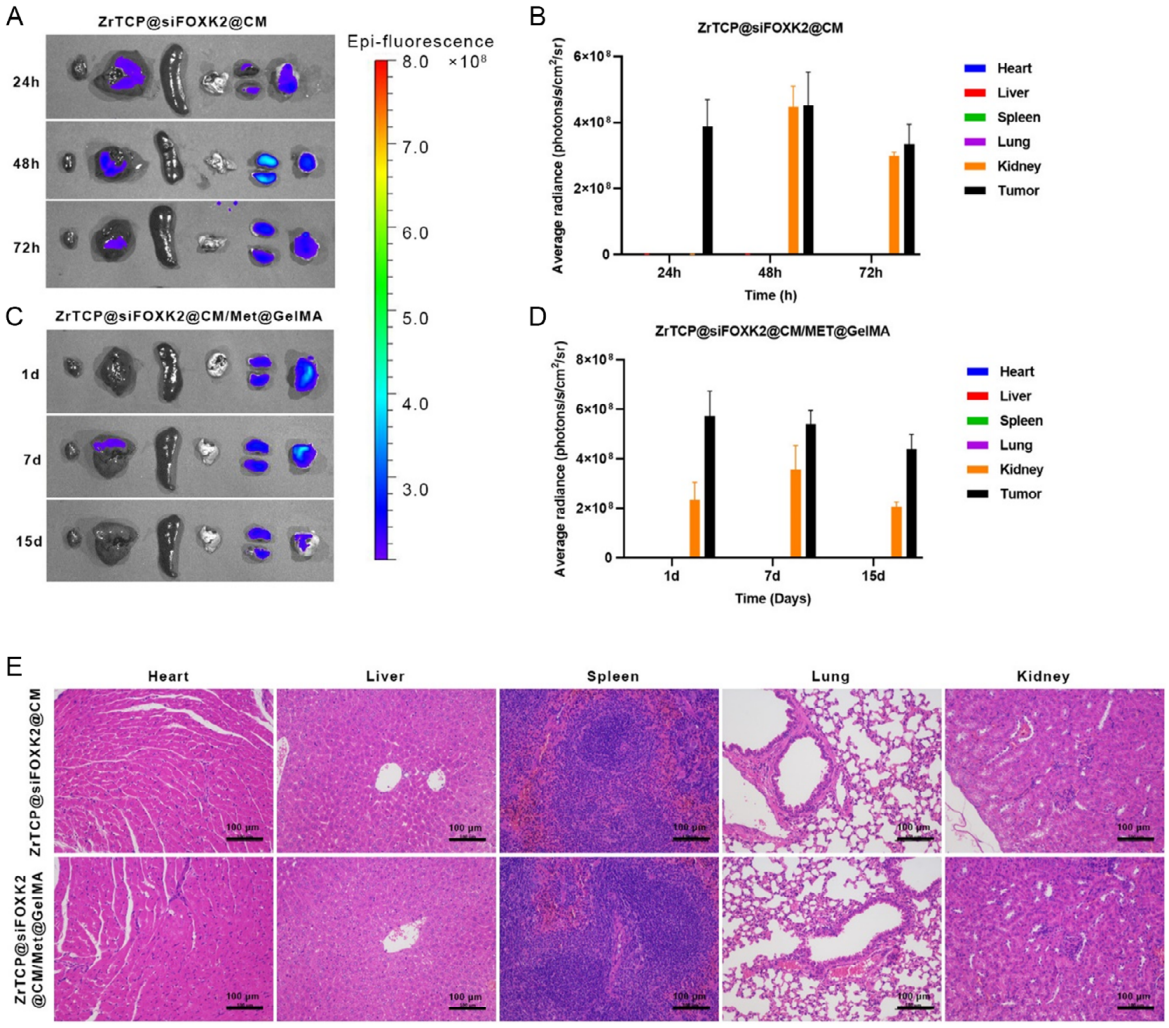
Biodistribution and organotoxicity in a nude mouse xenograft tumor model. A–D) Fluorescence imaging of main organs (L–R: heart, liver, spleen, lung, kidney, tumor) in tumor‐bearing nude mice post‐intravenous injection of ZrTCP@siFOXK2@CM (A,B) and post‐intratumoral injection of ZrTCP@siFOXK2@CM/Met@GelMA (C,D) using the in vivo imaging system. E) Hematoxylin and eosin (H&E)‐stained sections of main organs from tumor‐bearing nude mice 7 days after intravenous injection of ZrTCP@siFOXK2@CM and intratumoral injection of ZrTCP@siFOXK2@CM/Met@GelMA (*n* = 3, scale bar: 100 μm). Data in (B,D) are presented as the mean ± S.E.M. from three independent experiments, statistical significance among groups was determined by one‐way ANOVA, *P < 0.05, **P < 0.01, ***P < 0.001, and ****P < 0.0001.

Continuing from our previous findings, we delved into evaluating the in vivo antitumor efficacy of our formulated ZrTCP@siFOXK2@CM/Met@GelMA GelMA hydrogel. Initially, using a mouse xenograft model, we assessed the in vivo distribution and organ toxicity of both ZrTCP@siFOXK2@CM NPs and the ZrTCP@siFOXK2/Met@CM@GelMA microgel. As evident from **Figure** **6**A,B, the in vivo imaging of small animals post tail vein injection of 100 μL of 5 mg mL^−1^ ZrTCP@siFOXK2@CM NPs revealed a substantial NP aggregation in tumor tissues within a mere 24 h. Concurrently, significant NPs were also detected in the liver and kidneys of the mice, with a persistent presence even after 72 h. In stark contrast (Figure 6C,D), when the tumor tissue was directly injected with an equivalent amount of ZrTCP@siFOXK2/Met@CM@GelMA microgel, the signal from ZrTCP@siFOXK2@CM NPs was predominantly localized within the tumor tissue, enduring for at least 15 days.

Subsequently, mice injected with ZrTCP@siFOXK2@CM NPs for 72 h and those administered with ZrTCP@siFOXK2/Met@CM@GelMA microgel intra‐tumorally for 15 days were euthanized to obtain primary organ (heart, liver, spleen, lungs, kidneys) tissue samples. Histopathological examinations from hematoxylin and eosin (H&E)‐stained sections (Figure 6E) demonstrated that neither the tail vein injection of ZrTCP@siFOXK2@CM NPs nor the intratumoral injection of ZrTCP@siFOXK2/Met@CM@GelMA microgel‐induced any significant pathological damage in the major organs. This data confirms that tail vein injection of ZrTCP@siFOXK2@CM NPs, apart from tumor tissues, results in aggregation in both the liver and kidneys. In contrast, in situ injection of the ZrTCP@siFOXK2/Met@CM@GelMA microgel into the tumor displayed some degree of aggregation in kidney tissues, though without evident damage to the primary organs (heart, liver, spleen, lungs, kidneys) in either scenario.

### 2.7 Antitumor Efficacy of the ZrTCP@siFOXK2@CM@GelMA Microgel in Ovarian Cancer Models

Expanding on our previous observations, we further explored the in vivo antitumor efficacy of ZrTCP@siFOXK2@CM NPs and ZrTCP@siFOXK2/Met@CM@GelMA gel using the SKOV3 xenograft model in nude mice. Mice bearing SKOV3 xenografts were grouped into eight distinct categories, with each group undergoing a different treatment regimen as outlined: Group 1: mice received a tail vein injection of 100 μL PBS on the first day. Group 2: a tail vein injection of metformin in PBS at 0.2 mg kg^−1^ was administered on the first day. Groups 3 and 6: a tail vein injection of a mixture solution of ZrTCP@siFOXK2@CM NPs and metformin was administered on the first day. Groups 4 and 7: an intratumoral injection of ZrTCP@siFOXK2/Met@GelMA gel at 5 mg kg^−1^ was given on the first day. Groups 5 and 8: an intratumoral injection of ZrTCP@siFOXK2/Met@CM@GelMA gel at 5 mg kg^−1^ was given on the first day. From the second day onward, Groups 6–8 underwent in situ laser irradiation (650 nm, 0.25 W cm^−2^ for 5 min) every other day. Mice were monitored bi‐daily for weight and tumor growth, with euthanization occurring when the maximum tumor diameter reached 2 cm. Following euthanization, tumor and primary organ tissues (heart, liver, spleen, lungs, kidneys) were harvested for histological (H&E) and immunohistochemical examination.

**Figure 7:**
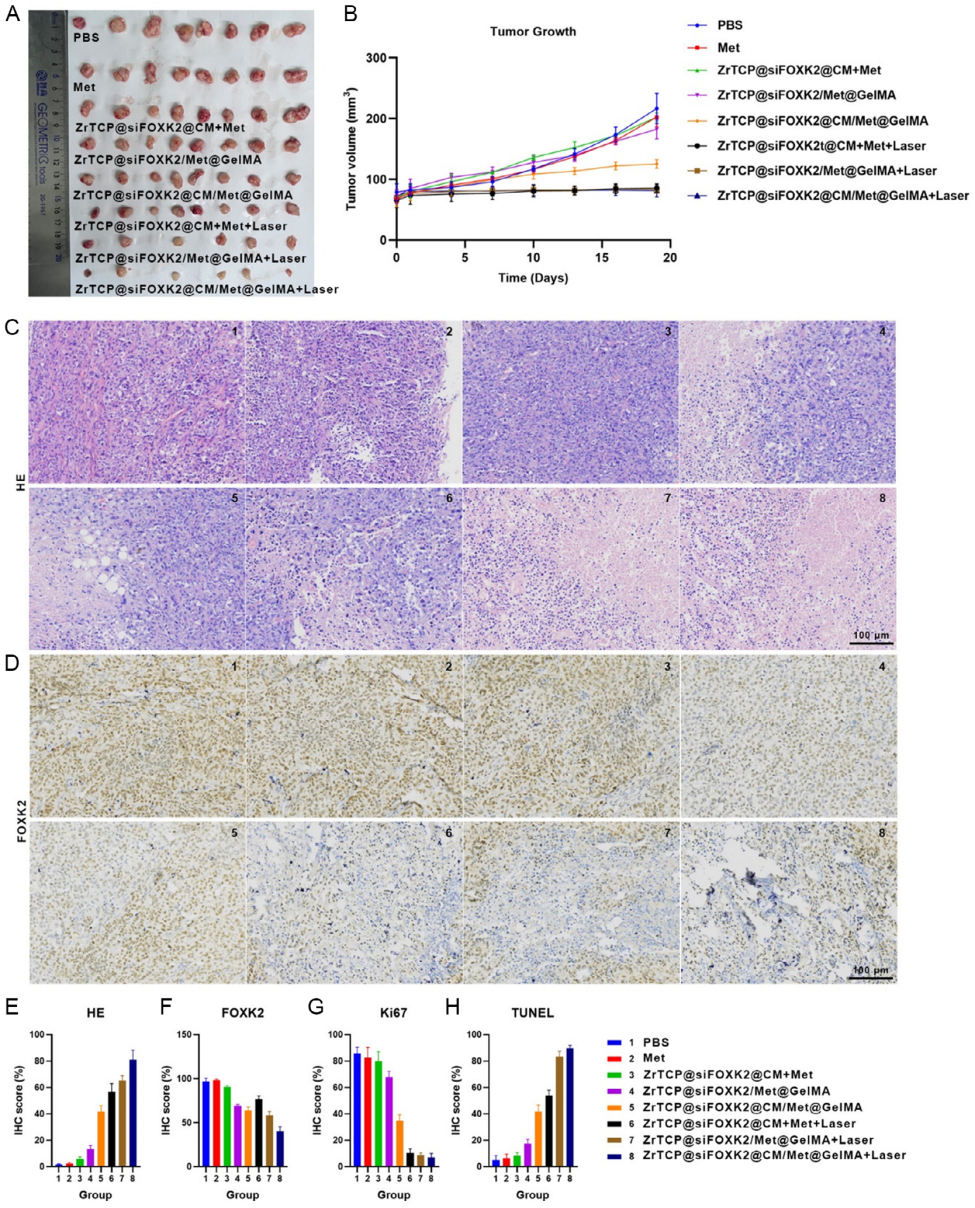
Antitumor effects of ZrTCP@siFOXK2@CM NPs and ZrTCP@siFOXK2@CM/Met@GelMA microgel in SKOV3 xenograft tumor model. A) Photos of tumors from SKOV3‐xenograft‐bearing nude mice post 18 days of various treatments, alongside B) the tumor growth curve (*n* ≥ 6). C) H&E sections and D) FOXK2 immunohistochemistry (IHC) staining of tumor tissues from SKOV3‐xenograft‐bearing nude mice following different treatments (*n* = 3; scale bar: 100 μm). E–H) IHC scores for tumor tissue staining: E) H&E, F) FOXK2, G) Ki67, and H) TUNEL (*n* = 3). Data in (B,E–H) are presented as the mean ± S.E.M. from three independent experiments; statistical significance among groups was determined by one‐way ANOVA, *P < 0.05, **P < 0.01, ***P < 0.001, and ****P < 0.0001.

As depicted in **Figure** **7**A,B, when compared to the PBS control and the metformin injection groups, the ZrTCP@siFOXK2/Met@CM@GelMA (Group 5), ZrTCP@siFOXK2/Met@CM+laser (Group 6), ZrTCP@siFOXK2/Met@GelMA+laser (Group 7), and ZrTCP@siFOXK2/Met@CM@GelMA+laser (Group 8) groups all exhibited significant inhibition of tumor growth. Notably, the tumor growth inhibition capability of Groups 6–8 was markedly superior to that of Group 5, but no discernible differences were observed among them. H&E results (Figure 7C) indicated varying degrees of necrosis in tumor tissues from Groups 3–8. Evaluated by necrotic area, the percentage of tumor necrosis was significantly higher in Groups 5–8, with respective figures of 45%, 55%, 65%, and 80%, in stark contrast to the 12% observed in Group 4 (Figure 7E). Furthermore, immunohistochemical findings for tumor tissues revealed a substantial decline in the proliferation index Ki67 in Groups 4–8 (Figure 7G and S7, Supporting Information) coupled with a significant increase in the apoptosis index terminal deoxynucleotidyl transferase dUTP nick end labeling (TUNEL) (Figure 7H and S8, Supporting Information) when juxtaposed with Groups 1–3. Additionally, tumor tissue FOXK2 gene expression levels were assessed (Figure 7D,F). Using the PBS‐treated group as the benchmark (100%), FOXK2 gene expression in Groups 3–8 was 90%, 65%, 60%, 70%, 58%, and 40%, respectively. In comparison with control Groups 1 and 2, a significant reduction in FOXK2 gene expression was observed across Groups 3–8.

Collectively, these findings underscore the capability of combined treatments—tail vein injection of ZrTCP@siFOXK2 @CM NPs and metformin mixture with laser therapy, intratumoral injections of either ZrTCP@siFOXK2/Met@GelMA with laser or ZrTCP@siFOXK2/Met@CM@GelMA with or without laser—to notably inhibit tumor growth and induce tumor tissue apoptosis. Moreover, tail vein injection of ZrTCP@siFOXK2@CM NPs or intratumoral injections of ZrTCP@siFOXK2/Met@GelMA or ZrTCP@siFOXK2/Met@CM@GelMA effectively diminished the FOXK2 gene expression in tumor tissues.

## 3 Discussion

The challenge of managing ovarian cancer is magnified by its heterogeneity, its tendency to remain asymptomatic in initial stages, and the evolution of chemoresistance during the therapeutic course. These barriers emphasize the pressing need for refined therapeutic strategies targeting the unique susceptibilities of ovarian cancer cells. A burgeoning body of research has pinpointed metabolic reprogramming as a distinct hallmark of cancer cells, opening avenues for specialized therapeutic interventions.^[^ ^43^, ^44^, ^45^ ^]^

The antidiabetic medication, metformin, has garnered attention for its promising anticancer capabilities.^[^ ^46^ ^]^ Its mechanism, notably through modulating mitochondrial oxidative phosphorylation and orchestrating the AMPK signaling pathway, offers intriguing therapeutic possibilities.^[^ ^47^ ^]^ Our findings robustly suggest that the antiproliferative prowess of metformin is heightened when combined with FOXK2 siRNA. The forkhead box (FOX) family of transcription factors, with FOXK2 in particular, stands out as instrumental in metabolic reprogramming and resilience under metabolic duress.^[^ ^18^, ^19^, ^48^ ^]^ These features nominate them as compelling therapeutic contenders. Yet, bridging the chasm between in vitro observations and in vivo therapeutic triumphs demands an astute approach to drug delivery.

The quandaries inherent to the targeted conveyance of therapeutic agents, especially siRNAs, are well chronicled. These nucleic acid derivatives often grapple with obstacles like rapid degradation, hindered cellular entry, and erratic biodistribution.^[^ ^26^, ^49^ ^]^ Our innovative methodology encases FOXK2 siRNA within biomimetic ZrTCP NPs, and these loaded NPs are subsequently incorporated alongside metformin into GelMA microspheres. This approach directly addresses the mentioned challenges. Existing literature extols the virtues of NPs for superior cellular entry and amplified therapeutic potential, particularly for siRNA delivery.^[^ ^50^ ^]^ The biomimetic design of our NPs augments tumor specificity, thereby curtailing off‐target repercussions and systemic toxicity.^[^ ^51^ ^]^

In a pivotal innovation, our strategy harnesses near‐infrared light exposure to the TCPP component within the ZrTCP NPs, invoking a photodynamic reaction analogous to traditional photodynamic therapy.^[^ ^52^ ^]^ This therapeutic cascade culminates in the localized generation of ROS that can expedite cancer cell destruction. Merging this controllable, dynamic tactic with our distinctive therapeutic concoction substantially bolsters cancer cell mortality rates.

In summary, the intricate synergy of metformin, FOXK2 siRNA, and the TCPP‐activated photodynamic capabilities of ZrTCP NPs formulates a comprehensive strategy. This approach adeptly targets the metabolic vulnerabilities of ovarian cancer cells while fine‐tuning the precision of drug delivery. Our findings, though promising, underscore the necessity for deeper exploration. This includes recognizing the current study's limitations, particularly in safety assessment. The lack of extensive long‐term toxicity data and in‐depth blood chemistry analysis marks a significant oversight. Future research endeavors should focus on these areas, integrating thorough long‐term toxicity studies and detailed blood chemistry evaluations. Such comprehensive investigations are pivotal not only for a complete understanding of the ZrTCP NPs’ safety profile but also for refining dosing protocols, uncovering potential resistance mechanisms, and anticipating adverse reactions, thereby enhancing the clinical efficacy and safety of our approach.

## 4 Conclusion

Our research presents a cutting‐edge therapeutic strategy tailored for ovarian cancer treatment. The convergence of metformin, FOXK2 siRNA, and light‐activated ZrTCP NPs encapsulated within GelMA microspheres offers an unprecedented targeting precision. This holistic approach not only addresses the metabolic vulnerabilities of the tumor, but also ensures the targeted delivery of therapeutic agents, maximizing their efficacy while minimizing collateral damage. Future studies and clinical trials could further refine this method, making it a frontline therapy for ovarian cancer and potentially revolutionizing the landscape of cancer therapeutics.

## 5 Experimental Section

The experimental methods and main materials are detailed in Supporting Information.

5.1

5.1.1

##### Animal Experiments

All animal experiments were conducted in compliance with the guidelines and protocols of the Institutional Animal Care and Use Committee of China and were approved by the Institutional Animal Care and Use Committee of The Chinese University of Hong Kong, Shenzhen (No. CUHKSZ‐AE2021013). Additionally, all experimental procedures also adhered to the European Union's respective guidelines for the accommodation and care of animals.

## Conflict of Interest

The authors declare no conflict of interest.

## Supporting information

Supplementary Material

## Data Availability

The data that support the findings of this study are available from the corresponding author upon reasonable request.
