# Supplementary material for: Exploiting the Warburg Effect: Co‐Delivery of Metformin and FOXK2 siRNA for Ovarian Cancer Therapy
Source: Small Sci. 2024 Jan 29;4(3):2300192. doi: 10.1002/smsc.202300192 (PMC11935110; doi:10.1002/smsc.202300192)
Supplement: Supplementary file 1 — Supplementary Material [file SMSC-4-2300192-s001.pdf]

Supporting Information

Exploiting the Warburg Effect: Co-Delivery of Metformin and *FOXK2* siRNA for Ovarian Cancer Therapy

Wenhui Zhou, Xiaodong Ma, Jianpeng Xiao, Xiaohui He, Chang Liu, Xiaoyu Xu, Tapani Viitala, Jing Feng, and Hongbo Zhang**

Experimental Section/Methods

Materials: Zirconium(IV) oxychloride octahydrate ($ZrOCl_2 \cdot 8H_2O$, Cat: V900193), Metformin (Cat: 317240,) and the photosensitizer TCPP (Cat: 257613) were purchased from Sigma-Aldrich. Gelatin was purchased from Aamas (Shanghai, China). siCtrl, siFOXK2 (sense: 5'-CCGAGCACAAACAUCAAGATT-3'; antisense: 5'-UCUUGAUGUUUGUGCUCGGTT-3') and siFOXK2-Cy5.5 were purchased from Sangon Biotech (Shanghai) Co., Ltd.,. Cell lines SKOV3 and OVCAR3 were obtained from the American Type Culture Collection (ATCC), and maintained in DMEM medium (Gibco) supplemented with 10% FBS and 1% PS.

Mamosphere formation assay: The cells were first treated with 100 nM siRNA and/or 20-30 mM Metformin for 24 hours. Next, the cells were seeded into 6-well ultra-low attachment plates at the density of 5000 cells/well. The cells were maintained in DMEM medium supplemented with 20 ng/mL basic fibroblast growth factor (bFGF, Cat: 3718-FB, Bio-Techne), epidermal growth factor (EGF, Cat: SRP3027, Sigma-Aldrich), 2% B27 (Cat: 17504044, Thermo Fisher) and cultured at 37°C with 5% CO_2, and 200 μl of fresh medium was added to each well. Fourteen days later, spheres greater than 50 μm were countered.

Synthesis of ZrTCP NPs: ZrTCP NPs were synthesized by incorporating the photosensitizer TCPP and Zr^{4+} according to the previous protocol[1-3]. Briefly, 300 mg $ZOrCl_2 \cdot 8H_2O$, 100 mg TCPP and 2.2 g benzoic acid were dissolved in 100 mL DMF and heated to 95℃ under stirred condition for 5 hours. Subsequently, the solution was cooled down to room temperature and centrifuged at 15,000 rpm for 15 minutes to collect the ZrTCP NPs. The remaining chemicals were removed by washing with DMF, and the collected ZrTCP NPs were dried at 60 ℃. Finally, the ZrTCP NPs were dispersed in deionized water and ethanol

and characterized by transmission electron microscope (TEM) and dynamic light scattering (DLS).

siFOXK2 loading and synthesis of ZrTCP@siFOXK2@CM NPs:
The siRNA was incorporated by combining ZrTCPNPs with siRNA in DEPC water, then stirring at 4°C for an hour. Subsequently, ZrTCP@siFOXK2 NPs were separated using centrifugation at 15,000 rpm and 4°C for 15 minutes. The RNA concentration in the supernatant was measured to determine the loading capacity.
SKOV3 cells in the logarithmic growth phase were harvested and rinsed thrice with PBS. They were then incubated in tris buffer (comprising 10 mM tris, 10 mM $MgCl_2$, and a protease inhibitor) for an hour at 4°C. Following this, the cells underwent sonication for 10 minutes in an ice bath. Cell membrane fragments were then procured through differential centrifugation (500 g for 10 minutes, followed by 10,000 g for 10 minutes). The resultant aqueous solution was combined with ZrTCP@siFOXK2 NPs in deionized water at 4°C and stirred overnight. In the end, ZrTCP@siFOXK2@CM NPs coated with the cell membrane were acquired through centrifugation.

Encapsulation within GelMA Microspheres: Microfluidic chip technology was utilized to encapsulate ZrTCP@siFOXK2@CM NPs and Metformin within GelMA microspheres. An aqueous phase, comprising 1 mg/ml ZrTCP@siFOXK2@CM NPs, 300 mM Metformin, GelMA prepolymer, and a crosslinker, was introduced into a microfluidic device as an inner flow at a rate of 0.1 mL/min. Concurrently, another aqueous phase, which contained a 5% Span80 mineral oil solution, was introduced as an external flow. By adjusting the flow rate of the external phase, the microfluidic device was configured to produce ZrTCP@siFOXK2@CM/Met@GelMA microspheres. Subsequently, ultraviolet (UV) light irradiated the droplets, facilitating on-chip photopolymerization. The resultant GelMA microspheres were then harvested and rinsed with PBS. Additionally, the GelMA prepolymer solution was concocted in line with our established protocol[4].

Cellular Interaction Studies: ZrTCP@siFOXK2-Cy5.5 and ZrTCP@siFOXK2-Cy5.5@CM NPs were first prepared. Subsequently, SKOV3 and OVCAR3 cells were cultured and exposed to 15 μg/ml ZrTCP@siFOXK2-Cy5.5 or ZrTCP@siFOXK2-Cy5.5@CM NPs. After incubation for 2-6 hours, cellular uptake efficiency was assessed using confocal microscopy

and flow cytometry. Lysosome escape capability was determined by co-localization studies with lysosomal trackers.

Cytotoxicity and Photodynamic Properties: To evaluate the cytotoxic effects of ZrTCP@siFOXK2 and ZrTCP@siFOXK2@CM NPs when combined with Metformin, SKOV3 and OVCAR3 cells were cultured under standard conditions. Once reaching desired confluency, cells were treated with 15 μg/ml ZrTCP@siFOXK2 or ZrTCP@siFOXK2@CM NPs in combination with 0-30mM Metformin. The next day, cells were exposed to infrared light with a wavelength of 650 nm at an intensity of 50 W/cm^2 for a duration of 5 minutes. After 48 hours of treatment, cell viability was determined using the CCK-8 assay, following the manufacturer's instructions.

For photodynamic studies, post-treatment, cells were exposed to infrared light with a wavelength of 650 nm at an intensity of 50 W/cm^2 for a duration of 5 minutes. Subsequent to this light exposure, cells were returned to the incubator for an additional 4 hours to allow for potential photodynamic effects to manifest. To probe the generation of reactive oxygen species (ROS), cells were incubated with specific fluorescence probes tailored to detect ROS. Fluorescence intensity was then quantified using a confocal microscope, indicative of ROS levels within the cells.

For insights into the activation of the AMPK signaling pathway, cells were lysed and subjected to Western blotting. Specific primary antibodies against key proteins of the AMPK pathway were used, followed by appropriate secondary antibodies. Protein bands were visualized using enhanced chemiluminescence, and band intensities were quantified to determine the extent of pathway activation.

In Vivo Studies: All animal experiments were conducted in compliance with the guidelines and protocols of the Institutional Animal Care and Use Committee of China and were approved by the Institutional Animal Care and Use Committee of The Chinese University of Hong Kong, Shenzhen (No. CUHKSZ-AE2021013). Additionally, all experimental procedures also adhered to the European Union’s respective guidelines for the accommodation and care of animals. Nude mice were used to establish SKOV3 tumor xenograft models. Approximately 1 x 10^6 SKOV3 cells in 100 μL of PBS were subcutaneously injected into the right flank of each mouse. Once tumors reached a volume of approximately 100 mm^3, the mice were randomly assigned into eight distinct groups as follows: Group 1: Mice received a tail vein injection of 100μl PBS on the first day. Group 2:

A tail vein injection of metformin dissolved in PBS at a concentration of 0.2mg/kg was administered on the first day. Groups 3 and 6: On the first day, a tail vein injection of a solution containing both 5mg/kg ZrTCP@siFOXK2@CM NPs and 0.2mg/kg metformin was administered. Groups 4 and 7: On the first day, an intratumoral injection of ZrTCP@siFOXK2/Met@GelMA at a concentration of 5mg/kg (concentration of ZrTCP@siFOXK2@CM NPs) was administered. Groups 5 and 8: Mice received an intratumoral injection of ZrTCP@siFOXK2/Met@CM@GelMA gel at 5mg/kg (concentration of ZrTCP@siFOXK2@CM NPs) on the first day. Starting from the second day, mice in groups 6, 7, and 8 were exposed to in situ laser irradiation with a wavelength of 650 nm at an intensity of 0.25W/cm^2. This exposure lasted for 5 minutes and was repeated every other day. Every other day, all the mice were monitored for both body weight and tumor growth. The monitoring continued until the tumor diameter in any mouse reached the threshold of 2cm. Upon reaching this threshold, the respective mouse was humanely euthanized. Post-euthanization, tumors were surgically excised and weighed. Furthermore, primary organ tissues, including the heart, liver, spleen, lungs, and kidneys, were harvested for subsequent analyses.

Immunohistochemical Examination: For immunohistochemical examination, tissues were fixed in 10% buffered formalin, embedded in paraffin, and sectioned. Routine histopathology was assessed using hematoxylin and eosin (H&E) staining. Immunostaining was performed on sections using specific antibodies against Ki67, TUNEL, and *FOXK2*, following standard protocols for antigen retrieval, blocking, and detection. Images were captured under a light microscope to assess cellular morphology and protein expression patterns.

Biodistribution and Safety Assessment: SKOV3 tumor-bearing mice received either a tail vein injection of ZrTCP@siFOXK2@CM NPs at a concentration of 5mg/kg or an intratumoral injection of ZrTCP@siFOXK2@CM@GelMA Microgel at the same concentration. The in vivo distribution of ZrTCP@siFOXK2@CM NPs was monitored using a small animal in vivo imaging system. The fluorescence signal from TCPP was captured to determine the nanoparticle distribution in the mice. Major organs, including the heart, liver, spleen, lungs, and kidneys, were subsequently excised and imaged to assess the nanoparticle accumulation in each organ. Additionally, the major organs were harvested, fixed, and sectioned for histological analysis using hematoxylin and eosin staining to evaluate any pathological alterations.

Statistical Analysis: Data were expressed as mean ± standard deviation (SD). Statistical analysis was performed using the Student's t-test, with a p-value of less than 0.05 considered statistically significant.

Figure S1. Cell viabilities of SKOV3 and OVCAR3 after treatment of metformin for 48 hours.

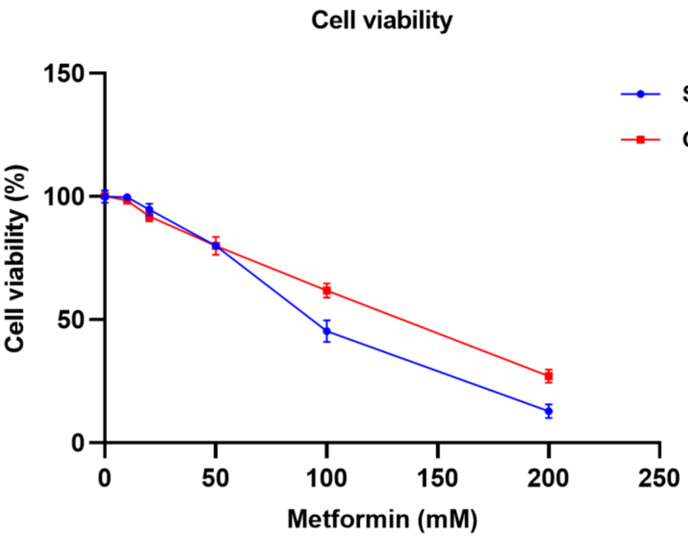

Figure S2. Cell viabilities of SKOV3 and OVCAR3 after treatment of siFOXK2 for 48 hours.

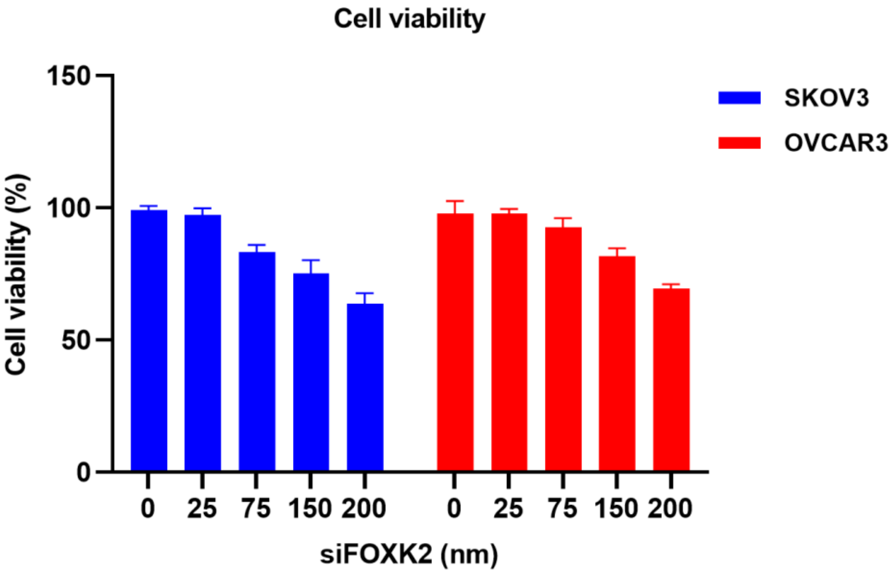

Figure S3. Cell viabilities of HEK293 and C3H10 after treatment of ZrTCP NPs for 48 hours.

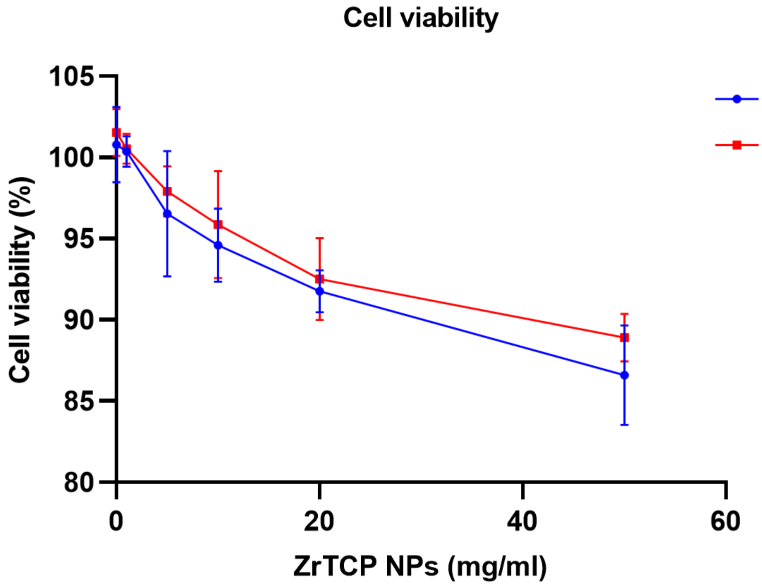

Figure S4. Cy5.5-positive SKOV3 cells post-incubation with ZrTCP@siFOXK2-Cy5.5 and ZrTCP@siFOXK2-Cy5.5@CM NPs, analyzed by flow cytometry.

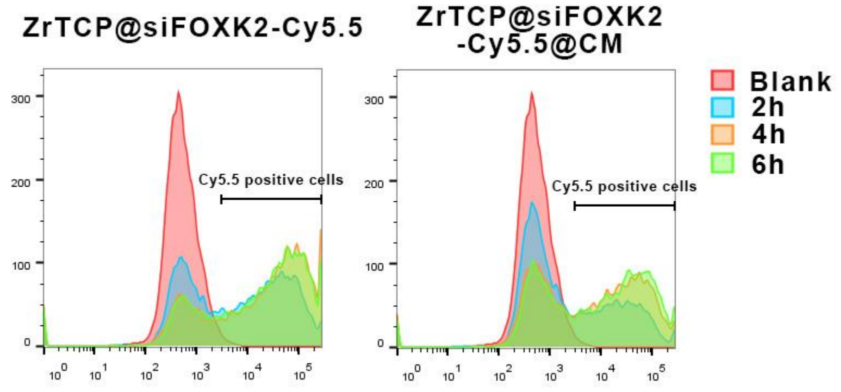

Figure S5. AMPK signal activation measured by western blot assay following various treatments for 48 hours.

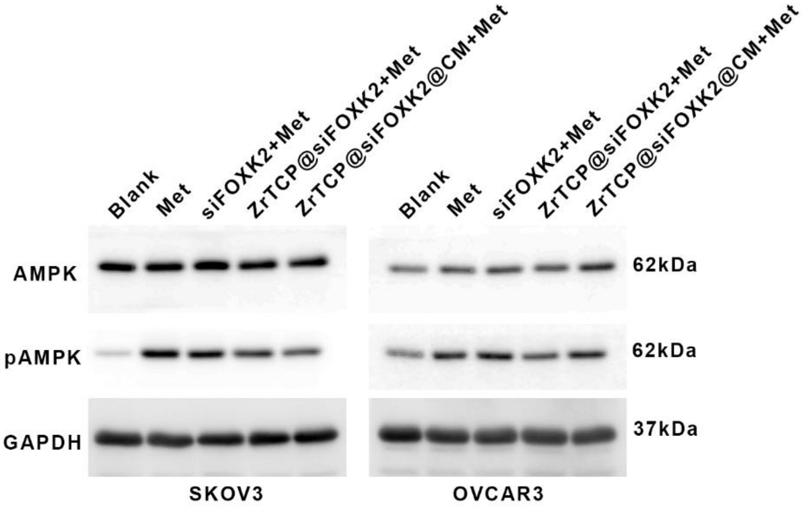

Figure S6. Representative confocal microscopy images of DCFH-DA stained SKOV3 cells (green: DCFH-DA; blue: Hoechst; scale bar: 20 μm).

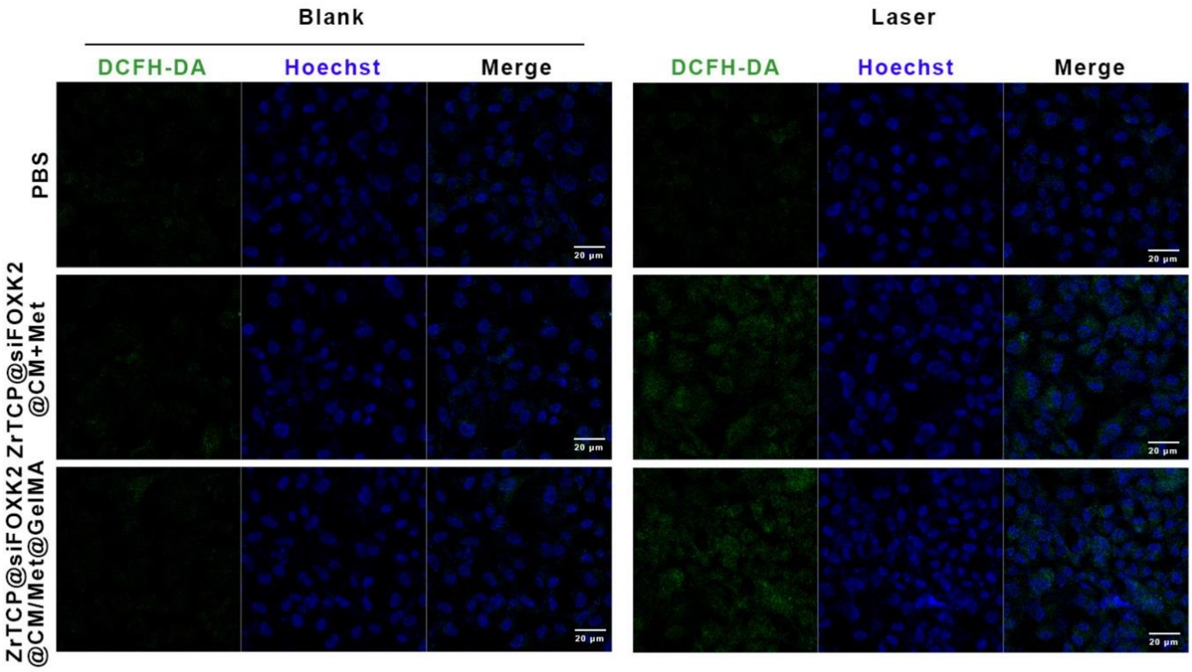

Figure S7. Ki67 staining of tumor tissues from SKOV3 xenograft-bearing nude mice following different treatments (n = 3; scale bar: 100 μm).

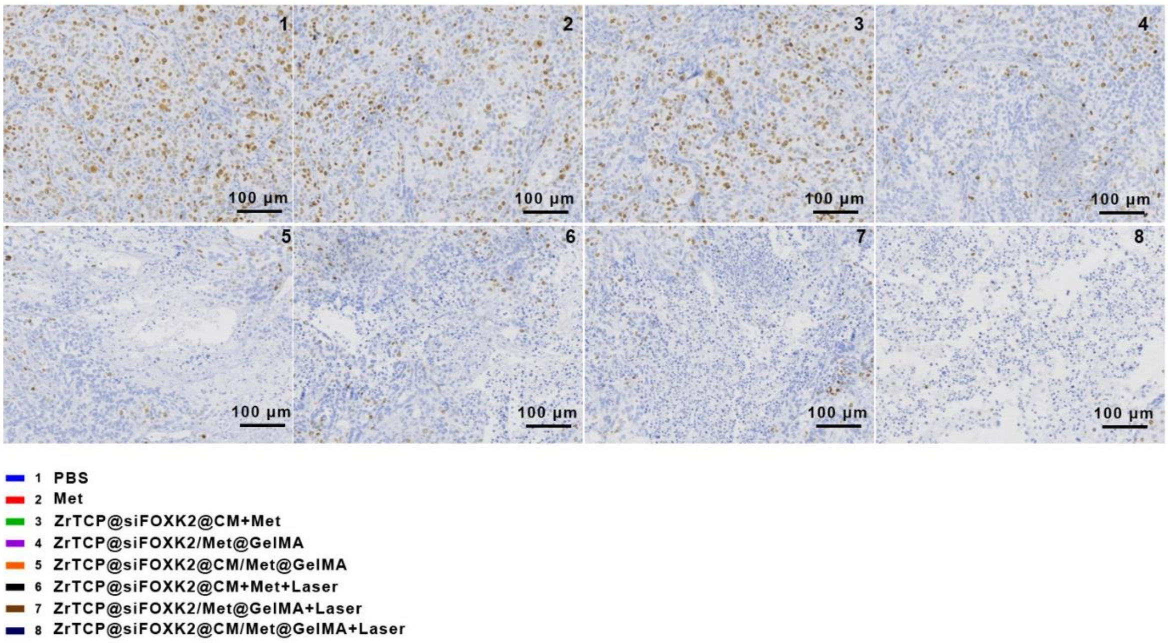

Figure S8. TUNEL staining of tumor tissues from SKOV3 xenograft-bearing nude mice following different treatments (n = 3; scale bar: 100 μm).

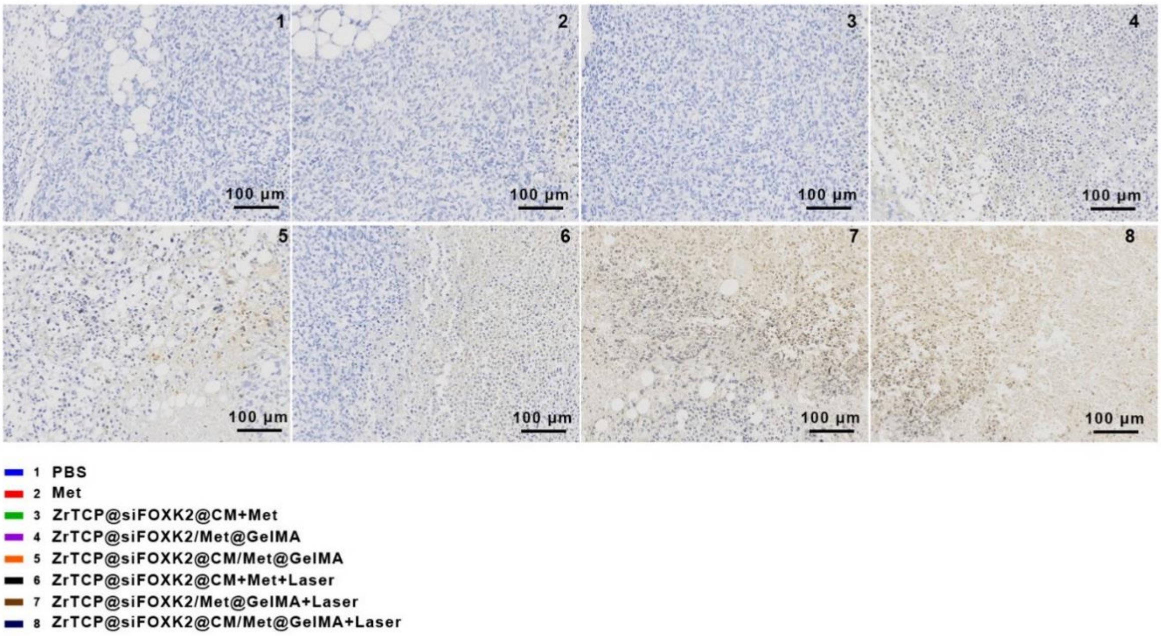

References

[1] W. L. Pan; Y. Tan; W. Meng; N. H. Huang; Y. B. Zhao; Z. Q. Yu; Z. Huang; W. H. Zhang; B. Sun; J. X. Chen, *Biomaterials* **2022,** *283*, 121449. DOI 10.1016/j.biomaterials.2022.121449.

[2] L. Yang; P. Cai; L. Zhang; X. Xu; A. A. Yakovenko; Q. Wang; J. Pang; S. Yuan; X. Zou; N. Huang; Z. Huang; H. C. Zhou, *J Am Chem Soc* **2021,** *143* (31), 12129-12137. DOI 10.1021/jacs.1c03960.

[3] Y. Ding; H. Xu; C. Xu; Z. Tong; S. Zhang; Y. Bai; Y. Chen; Q. Xu; L. Zhou; H. Ding; Z. Sun; S. Yan; Z. Mao; W. Wang, *Adv Sci (Weinh)* **2020,** *7* (17), 2001060. PMC7507500, DOI 10.1002/advs.202001060.

[4] X. Ma; W. Zhou; R. Zhang; C. Zhang; J. Yan; J. Feng; J. M. Rosenholm; T. Shi; X. Shen; H. Zhang, *Mater Today Bio* **2023,** *20*, 100663. PMC10232889, DOI 10.1016/j.mtbio.2023.100663.
